# Investigating the Anti-Inflammatory Effects of RCI001 for Treating Ocular Surface Diseases: Insight Into the Mechanism of Action

**DOI:** 10.3389/fimmu.2022.850287

**Published:** 2022-03-24

**Authors:** Seunghoon Kim, Ye Won Jang, Young-ah Ku, Yungyeong Shin, Md Mahbubur Rahman, Myung-Hee Chung, Yong Ho Kim, Dong Hyun Kim

**Affiliations:** ^1^RudaCure Co. Ltd., Incheon, South Korea; ^2^Gachon Pain Center and Department of Physiology, Gachon University College of Medicine, Incheon, South Korea; ^3^Department of Ophthalmology, Gil Medical Center, Gachon University College of Medicine, Incheon, South Korea

**Keywords:** RCI001, ocular surface disease (OSD), inflammation, Rac1, NLRP3 inflammasome

## Abstract

The ocular surface is continuously exposed to various environmental factors, and innate and adaptive immunity play crucial roles in ocular surface diseases (OSDs). Previously, we have reported that the topical application of RCI001 affords excellent anti-inflammatory and antioxidant effects in dry eye disease and ocular chemical burn models. In this study, we examined the inhibitory effects of RCI001 on the Rac1 and NLRP3 inflammasomes *in vitro* and *in vivo*. Following RCI001 application to RAW264.7 and Swiss 3T3 cells, we measured Rac1 activity using a glutathione-S-transferase (GST) pull-down assay and G-protein activation assay kit. In addition, we quantified the expression of inflammatory cytokines (interleukin [IL]-1β, IL-6, and tumor necrosis factor [TNF]-α) in lipopolysaccharide (LPS)-stimulated RAW264.7 cells using ELISA and real-time PCR. In the mouse ocular alkali burn model, RCI001 was administered *via* eye drops (10 mg/mL, twice daily) for 5 days, and 1% prednisolone acetate (PDE) ophthalmic suspension was used as a positive control. Corneal epithelial integrity (on days 0-5) and histological examinations were performed, and transcript and protein levels of Rac1, NLRP3, caspase-1, and IL-1β were quantified using real-time PCR and western blotting in corneal tissues collected on days 3 and 5. We observed that RCI001 dose-dependently inhibited Rac1 activity and various inflammatory cytokines in LPS-stimulated murine macrophages. Furthermore, RCI001 restored corneal epithelial integrity more rapidly than corticosteroid treatment in chemically injured corneas. Compared to the saline group, activation of Rac1 and the NLRP3 inflammasome/IL-1β axis was suppressed in the RCI001 group, especially during the early phase of the ocular alkali burn model. Topical RCI001 suppressed the expression of activated Rac1 and inflammatory cytokines *in vitro* and rapidly restored the injured cornea by inhibiting activation of Rac1 and the NLRP inflammasome/IL-1*β* axis *in vivo*. Accordingly, RCI001 could be a promising therapeutic agent for treating OSDs.

## Introduction

The ocular surface comprises the cornea, limbus, conjunctiva, and tear film and is continuously exposed to the external environment, including various antigens and pathogens (1). Its integrity is vital for the healthy functioning of the eye, and both innate and adaptive immunity play important parts in ocular surface diseases (OSDs). OSDs include dry eye disease (DED), meibomian gland dysfunction, allergic conjunctivitis, chemical burns, and immunological conditions, such as ocular graft-versus-host disease and Stevens-Johnson syndrome. DED, a representative OSD, is mainly characterized by tear film instability, tear hyperosmolarity, and ocular surface inflammation (2, 3). OSDs present a substantial public burden. In the United States, the annual medical costs per patient with dry eye are estimated to be $783, with an overall cost of approximately $3.84 billion per year (4). In addition, the total social cost, including the loss of productivity, was reportedly $55.4 billion per year (4). Topical corticosteroids, cyclosporin A, and tacrolimus are effective therapeutic agents against ocular surface inflammatory diseases. However, topical corticosteroids have clinical limitations due to potential risks, such as secondary glaucoma, infection, and cataract formation (5). It should be noted that non-corticosteroid agents are not as potent as corticosteroids and cannot modulate innate immunity, a key regulator of ocular surface inflammation (6, 7). The ROS-NLRP3-IL-1β signaling axis is found to be activated in human corneal epithelial cells in a hyperosmolar environment, as well as in the tear fluid of patients with DED (8). NLRP3 inflammasome is involved in OSD, and inhibition of the NLRP3 inflammasome pathway can reduce chronic ocular surface complications after external damage (9). Accordingly, OSDs present a significant unmet medical need considering currently available therapeutic agents. Thus, numerous studies have been conducted to develop novel therapeutic agents for OSD.

RCI001 is a novel therapeutic candidate for treating OSDs, including DED. 8-Oxo-2ʹ-deoxyguanosine (8-oxo-dG), the main component of RCI001, is produced during DNA repair *in vivo* (10). Previous studies have demonstrated that 8-oxo-dG has excellent anti-inflammatory and antioxidative effects in experimental inflammatory models (10–14). Pretreatment with 8-oxo-dG was found to efficiently suppress proinflammatory cytokines (tumor necrosis factor (TNF)-α, interleukin (IL)-6, IL-18, and IL-12) in the serum and neutrophil infiltration in an intraperitoneal lipopolysaccharide (LPS) injection-induced inflammation model (10). Additionally, 8-oxo-dG reportedly inhibits nitric oxide (NO) production and cyclooxygenase-2 (COX-2) activity in an intrastriatal LPS injection model while reducing transcript levels of proinflammatory cytokines (IL-1β, IL-6, and TNF-α) in activated BV2 cells (11). Moreover, 8-oxo-dG can modulate the production of reactive oxygen species (ROS) through NADPH oxidase, phagocytosis, and chemotaxis in stimulated murine macrophages and dose-dependently inhibits the secretion of interferon (IFN)-γ from macrophages (12). In ultraviolet (UV) B-irradiated hairless mice, 8-oxo-dG was shown to prevent UV-induced skin reactions and epidermal hyperplasia, and inhibit the activation of mitogen-activated protein kinases (MAPKs), activating transcription factor (ATF)-2, and c-Jun, as well as the increased matrix metallopeptidase (MMP)-9 and -13 expression (14). Furthermore, 8-oxo-dG reportedly suppressed allergy-associated immune responses and decreased airway resistance in ovalbumin-sensitized mice (15). It has been postulated that 8-oxo-dG mediates these broad anti-inflammatory, antioxidative, and anti-allergic effects by inhibiting Rac1, a GTPase involved in cell signaling (10, 12, 14, 15).

We have previously shown that RCI001 dose-dependently promoted the corneal healing and inhibited inflammation in ethanol-induced and alkali burn ocular injury models (5, 16). RCI001 suppressed neutrophil and macrophage infiltration in injured corneal tissues (5, 16). In addition, RCI001 was shown to improve ocular surface staining and tear secretion in mouse models of environmental- and inflammation-mediated DED (17). Given the broad anti-inflammatory effect of RCI001, we are currently developing RCI001 as a potential therapeutic agent against OSD. In the present study, we investigated the inhibitory effects of RCI001 on Rac1 and NLRP3 inflammasomes in murine macrophages (*in vitro*) and an ocular alkali burn model (*in vivo*).

## Materials and Methods

### Cell Culture

RAW 264.7, a murine macrophage cell line, was obtained from the American Type Culture Collection (Manassas, VA) and maintained in Dulbecco’s modified Eagle’s medium (DMEM), supplemented with 10% heat-inactivated fetal bovine serum (FBS), 2 mM L-glutamine, and 100 U/mL penicillin and streptomycin with incubation at 37°C in a 5% CO_2_ humidified incubator. On reaching the desired confluency, cells were seeded onto 6-cm dishes to perform the Rac1 activation assay, G-LISA activation assay, and *in vitro* enzyme-linked immunosorbent assay (ELISA)/real-time PCR for inflammatory cytokines. All experimental samples analyses are performed by 5 replicates (5 samples in each treatment group in each replicate), and mean data from each treatment group from each replicate was served as single data and used for statistical analysis. Each replicate was performed in different days.

### Rac1 Activation Assay (Pull-Down Assay)

Briefly, RAW264.7 cells were seeded in 90 mm Petri dishes. After overnight incubation, cells were treated with LPS (1 μg/mL) and RCI001 (1 μM) for 2 h. Active Rac1 was assessed using an Active Rac1 Detection kit (#8815, Cell Signaling Technology, USA). Then, the GST-PAK1-PBD fusion protein was deployed to bind the active GTP-bound Rac1, and western blot analysis was performed to examine the activated Rac1-GTP protein.

### Rac1 G-LISA Activation Assay

Briefly, Swiss 3T3 cells were seeded at a density of 2 × 105 cells/well in a 6-well plate. After overnight incubation, the medium was changed to DMEM containing 1% FBS for 24 h. Then, cells were transferred to serum-free DMEM for 24 h. Cells were treated with CN04 (1.0 μg/mL, Rac1 activator) and RCI001 (1~1000 nM) for 2 h. The Rac1 activity assay was performed according to the manufacturer’s protocol (BK128, Cytoskeleton Inc., USA).

### *In Vitro* ELISA

Briefly, RAW 264.7 cells were plated at a density of 8×104/well in a 48-well plate. After overnight incubation, the cells were stimulated with 1 μg/mL LPS for 2 h. Then, the cells were treated with RCI001. The supernatants were harvested at 24 h, and the concentration of IL-1β, IL-6, and TNF-α in each sample was detected using an ELISA kit (R&D Systems). All ELISA assessments were performed in accordance with the manufacturer’s instructions. IC_50_ values was estimated by non-linear regression analysis, using the following equation: Y=100/(1+(IC50/X)^HillSlope); where X is the concentration of RCI001; Y is the normalized response; and HillSlope is the Hill slope factor (Graphpad Prism Version 8, San Diego, CA, USA).

### Quantitative Real-Time PCR

Total RNA was isolated using an RNeasy Mini Kit (74104; Qiagen). Complementary DNA synthesis was performed using M-MLV Reverse Transcriptase (28025-013; Invitrogen) following the manufacturer’s instructions. The cDNA concentration was measured by qPCR using 2× iQ™ SYBR^®^ Green Supermix (Bio-Rad) to determine mRNA levels of IL-1β, IL-6, and TNF-α. cDNA was amplified as follows: 95°C (3 min), followed by 40 cycles of 95°C (15 s) and 60°C (40 s) using a CFX Connect™ real-time PCR detection system (Bio-Rad). To confirm the specificity, PCR products were subjected to melting curve analysis. The relative mRNA quantity in the experimental samples, compared with that in control samples, was determined using the comparative threshold method. Gene expression was normalized to that of GAPDH.

### Western Blotting

Briefly, the weighed tissue sample was added to NP-40 lysis buffer (EBA-1049, Elpis Biotech) containing protease inhibitor in accordance with the manufacturer’s instructions, followed by homogenization for 2 min. The proteins present in the lysate were quantified using a BCA protein assay kit (23227, Thermo Fisher Scientific). Equivalent amounts of protein were separated by sodium dodecyl sulfate-polyacrylamide gel electrophoresis (SDS-PAGE) and transferred to polyvinylidene fluoride membranes (ipvh00010, Millipore). Next, the blots were blocked, and the membrane was incubated with anti-IL-1β antibody (sc-7884, Santa Cruz), anti- IL-18 antibody (ab71495, Abcam), anti-caspase-1 antibody (sc-56036, Santa Cruz), anti-NLRP3 antibody (ab214185, Abcam), anti-Rac1 antibody (ab33186, Abcam), or anti-GAPDH antibody (sc-25778, Santa Cruz), overnight at 4°C, followed by horseradish peroxidase (HRP)-conjugated anti-rabbit or mouse IgG antibody for 1 h at room temperature. Enhanced chemiluminescence (ECL) reagents and a chemiluminescence analyzer (ImageQuant LAS 4000, GE Healthcare Life Sciences) were used to detect proteins.

### Animals and *In Vivo* Experimental Design

*In-vivo* experiments were performed using 8-week-old female ICR mice (a total 15) (Orient Bio Inc., Seongnam, Korea). Mice were maintained at the animal facility of Rudacure^®^, with free access to water and food. Anesthesia was induced by intraperitoneal injection of sodium pentobarbital (65 mg/kg) (Entobar, Hanlim Pharmaceuticals Co., Seoul, Korea). The right eye of each mouse was subjected to an ocular alkali burn, induced by applying a 2 mm piece of filter paper soaked in 0.1 N NaOH to the corneal center for 15 s, with the cornea then sufficiently rinsed with normal saline for 60 s. Mice were treated with the following ophthalmic solutions: normal saline, 1% prednisolone acetate (PDE) ophthalmic suspension (Predbell^®^, Chong Keun Dang Pharm, Seoul, South Korea), and 10 mg/mL RCI001. RCI001 was dissolved in a normal saline solution. After establishing the alkali burn model, the animals were equally divided in to three groups (n=5) and 5 μL of each solution was instilled into the right eye, twice daily for 5 days. Topical treatments were initiated 16–24 h after establishing the alkali burn model.

### Clinical Examinations

Corneal images were captured by a single observer (Y Shin) using a handheld slit-lamp biomicroscope (SL-17, Kowa, Tokyo, Japan) 5 days after chemical injury. Corneal epithelial integrity was evaluated by administering 1 drop of 3% Lissamine Green B (Sigma-Aldrich) to the inferior lateral conjunctival sac and photographed using a Dino-Lite digital camera (Dino-Lite Pro, AnMo Electronics Corp, Hsinchu, Taiwan). Evaluation of corneal epithelial integrity and corneal clarity were performed by three blinded observers by the scoring systems as described elsewhere (5, 17). Corneal epithelial integrity was scored using the following scale of 0 to 4 based on the intensity of corneal epithelial defects: 0 = no epithelial defect, 1 = less than 25% epithelial defect, 2 = 25–50% epithelial defect, 3 = 50–75% epithelial defect, and 4 = more than 75% epithelial defect (17). Corneal clarity was scored by following the scale from 0 to 4: 0, no opacity, completely clear cornea; 1=slightly hazy, iris and lens visible; 2=moderately opaque, iris and lens still detectable; 3=severely opaque, iris and lens hardly visible; and 4=completely opaque, with no view of the iris and lens.

### Histological Examination

After treatment, the eyeballs were enucleated, fixed in 10% formalin, and embedded in paraffin. Then, tissues were cut into 4-µm-thick sections and stained with hematoxylin and eosin (H&E). Next, the sections were incubated with a peroxidase blocking solution (Agilent, Santa Clara, CA, USA) for 10 min. Each section was counterstained with hematoxylin and mounted using Canada balsam. Finally, slides were observed and photographed under a light microscope (BX51 and DP72, Olympus, Japan). In the photographs of tissues with the H&E stain, corneal stromal thickness was quantified with ImageJ software (US National Institutes of Health) by 2 independent examiners.

### Statistical Analysis

Data were expressed as mean ± standard error of the mean (SEM). Differences between groups were evaluated by Bonferroni *post hoc* test following one-way ANOVA or Bonferroni *post hoc* test following two-way ANOVA. We analyzed the differences by using Prism 5.03 (GraphPad Software Inc., La Jolla, CA, USA). Statistical significance was set at p < 0.05.

## Results

### RCI001 Dose-Dependently Inhibited Rac1 Activity Without Affecting RhoA and Cdc42

**Figure 1:**
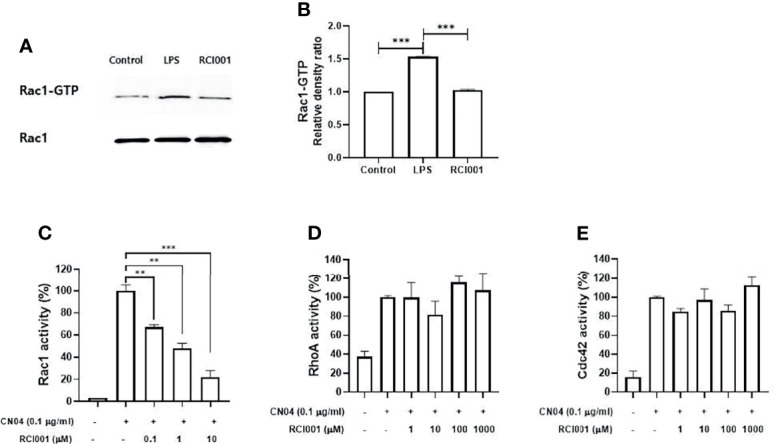
RCI001 suppressed the Rac1-GTP complex formation in lipopolysaccharide-stimulated RAW 264.7 **(A, B)**; and dose-dependently inhibited Rac1 activity **(C)** in CN04-stimulated Swiss 3T3 cells without affecting RhoA **(D)** and Cdc42 **(E)**. Data are means ± standard error of the mean (SEM). ** p<0.01, and ***p<0.001. LPS, RAW 264.7 cells stimulated with lipopolysaccharide; RCI001, application of RCI001 on lipopolysaccharide-stimulated RAW 264.7 cells.

Based on the results of the Rac1 pull-down assay, the Rac1-GTP complex formation was increased by 50% in LPS-stimulated Raw264.7 cells, and RCI001 inhibited this complex by 33% when compared to LPS-stimulated Raw264.7 cells (**Figures 1A, B**). In the Rac1 G-LISA activation assay, a notable dose-dependent inhibitory relationship could be observed between RCI001 and Rac1 activity. Rac1 activity was decreased by 52% and 78% following treatment with 1 and 10 µM RCI001, respectively (half-maximal inhibitory concentration [IC50] = 0.68 µM). RCI001 exhibited no inhibitory effects against RhoA and Cdc42, which belong to the same RhoA family as Rac1 (**Figures 1C–E**).

### RCI001 Dose-Dependently Inhibited Inflammatory Cytokines in LPS-Stimulated RAW 264.7 Cells

**Figure 2:**
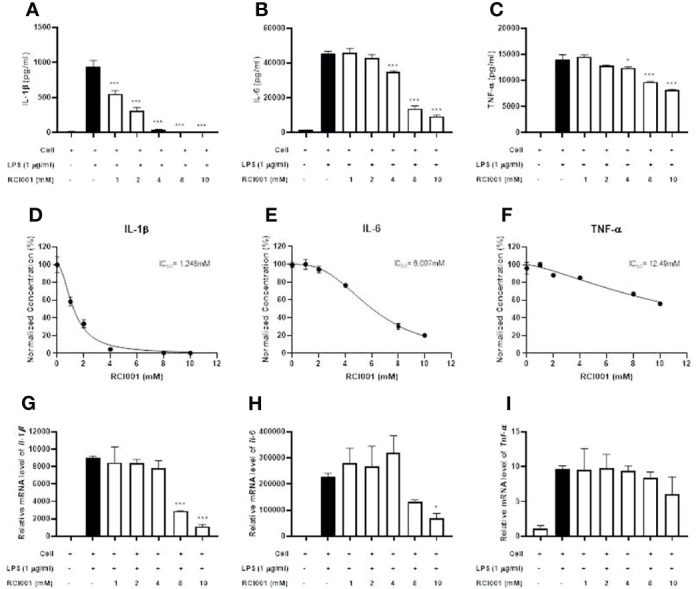
RCI001 dose-dependently inhibited inflammatory cytokines in LPS-stimulated RAW 264.7 cells. **(A–C)** Expression of IL-1β, IL-6 and TNF-α cytokines assayed by Enzyme-linked immunosorbent assay (ELISA). **(D–F)** measurement of IC50 value and **(G–I)** real-time reverse transcription-polymerase chain reaction (PCR). Data are means ± standard error of the mean (SEM). *p<0.05, and ***p<0.001 vs LPS group (Black bar). LPS, lipopolysaccharide.

Following LPS stimulation, levels of inflammatory cytokines (IL-1β, IL-6, and TNF-α) were significantly increased in RAW 264.7 cells; RCI001 decreased the production of IL-1β, IL-6, and TNF-α in LPS-stimulated RAW 264.7 cells (each p<0.05; IC50: 1.248 mM [IL-1β], 6.007 mM [IL-6], **Figures 2A–F**). In addition, RCI001 decreased transcript levels of IL-1β and IL-6, especially at a concentration exceeding 8 mM (each p<0.05, **Figures 2G, H**). The inhibitory effect of RCI001 on TNF-α was lower than that exhibited against IL-1 and IL-6 (**Figures 2C, I**).

### RCI001 Restored Injured Cornea Faster Than Corticosteroid Treatment and Clinically and Histologically

**Figure 3:**
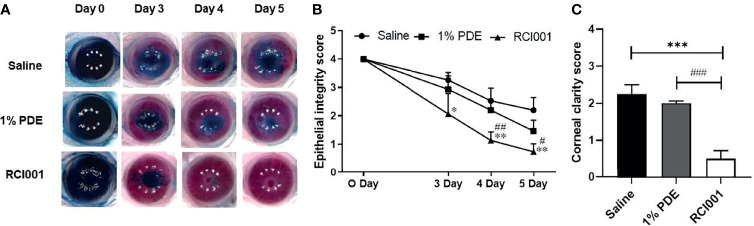
Therapeutic effects of RCI001 on corneal epithelial integrity and clarity in alkali burn induced ocular surface disease model. **(A)** Representative images of three groups from 0- 5 days. **(B)** Comparative analysis of corneal epithelial integrity score of three groups from 0- 5 days. **(C)** Comparative analysis of corneal clarity score of three groups on day 5. Data are reported as means SEMs (n = 10). *p<0.05, **p<0.01, and ***p<0.001 vs saline group; #p<0.05, ##p<0.01, and ###p<0.001 vs 1% PDE group, Bonferroni *post hoc* test following two-way ANOVA; **(B)** and Bonferroni *post hoc* test following one-way ANOVA **(C)** (n=5).

**Figure 4:**
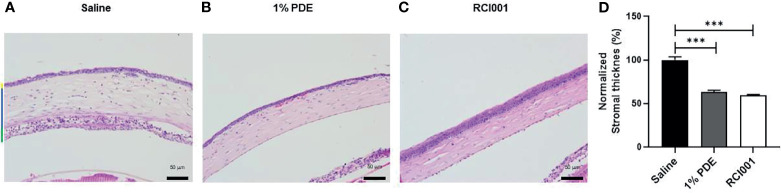
Therapeutic effects of topical RCI001 (8-oxo-dG) on histological changes of the cornea in alkali burn induced ocular surface disease model. **(A–C)** Representative images of hematoxylin and eosin (H&E) staining of the central cornea. **(D)** Comparison of stromal thickness of the central cornea. Yellow bar- epithelium, blue bar- stroma and green bar- endothelium whereas black bar indicates size 50 μm. Data are reported as means SEMs (n = 10). ***p < 0.001, Bonferroni *post hoc* test following one-way ANOVA.

On post-injury day 5, we observed superior and faster recovery of corneal epithelial defects in the RCI001 group than in the 1% PDE and saline groups (**Figures 3A, B**). It was observed that the corneal epithelial integrity score was significantly improved from day 0 to day 5 when compared with saline treated group. In addition, Corneal epithelial integrity score in RCI001 group were better than 1%PDE group from day 3 to 5 and there was significantly differed on day 4 and 5, indicating its therapeutic superiority (**Figure 3B**). The recovery of corneal clarity was greater in the RCI001 group than in other examined groups (**Figure 3C**). H&E staining revealed that edema in the corneal stroma was significantly reduced in the RCI001 and 1% PDE groups when compared with that in the saline group (**Figures 4A–C**). Compared to the 1% PDE and saline groups, corneal epithelial integrity and thickness recovered completely in the RCI001 group (**Figure 4D**).

### RCI001 Inhibited Activation of Rac1 and NLRP3-Caspase-1-IL-1β Pathway in the Early Phase of Ocular Alkali Burn Model

**Figure 5:**
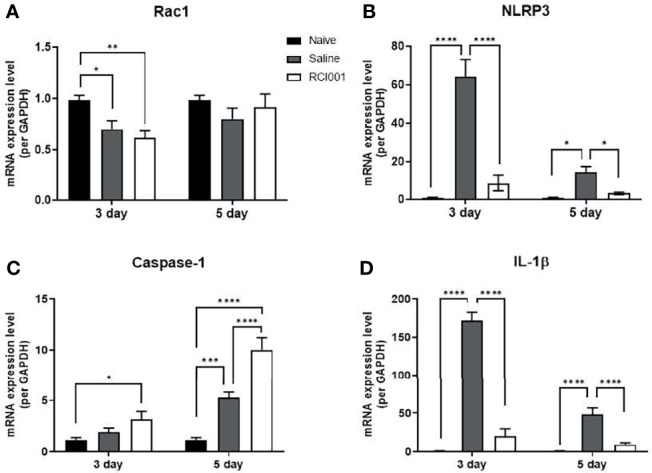
RCI001 inhibited activation of Rac1 and NLRP3-caspase-1-IL-1β pathway of ocular surface in the ocular alkali burn model measured by RT-PCR. Relative mRNA expression levels of Rac1 **(A)**, NLRP3 **(B)**, caspase-1 **(C)**, and IL-1β **(D)**. Data are presented as means ± standard error of the mean (SEM). *p<0.05, **p<0.01, ***p<0.001, and ****p<0.0001. IL-1β, interleukin-1β.

**Figure 6:**
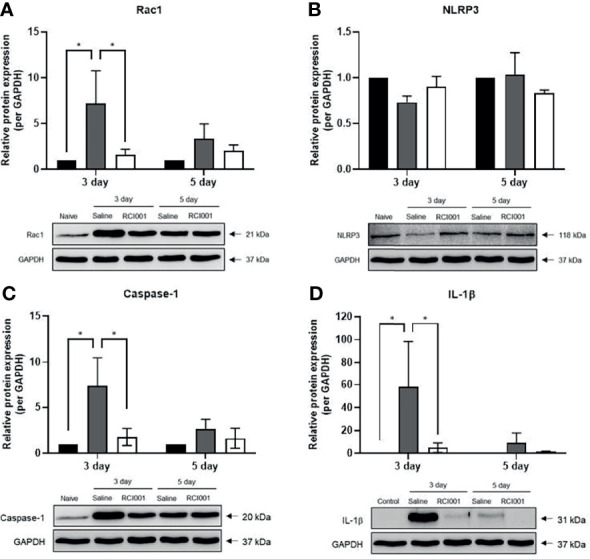
RCI001 inhibited activation of Rac1 and NLRP3-caspase-1-IL-1β pathway of ocular surface in the ocular alkali burn model measured by western blotting. Relative protein expression of Rac1 **(A)**, NLRP3 **(B)**, caspase-1 **(C)**, and IL-1β **(D)**. Data are presented as means ± standard error of the mean (SEM). *p<0.05. IL-1β, interleukin-1β.

In the excised cornea, the Rac1 transcript level was slightly reduced on day 3 after treatment in the RCI001 and saline groups when compared with those in the naïve group (p<0.01/<0.05), with no differences observed on day 5 post-treatment (**Figure 5A**). Compared to the saline group, Rac1 protein expression was considerably suppressed in the RCI001 group at day 3, but not day 5, post-treatment (p<0.05, **Figure 6A**). Transcript levels of NLRP3 and IL-1β were markedly increased in the saline group when compared to the naïve group (each p<0.0001); these levels were significantly decreased in the RCI001 group at day 3, similar to the naïve group (each p<0.0001) (**Figures 5B, D**). On day 5, the saline and RCI001 groups exhibited trends similar to those observed on day 3 (NLRP3: each p<0.05, IL-1β: each p<0.0001) (**Figures 5B, D**). However, NLRP3 protein levels did not significantly differ among the three groups (**Figure 6B**). IL-1β protein levels were reduced in the RCI001 group when compared with those in the saline group on day 3 (p < 0.05, **Figure 6D**); these levels did not significantly differ among the three groups on day 5. Meanwhile, the transcript level of caspase-1 was slightly increased in the RCI001 group at day 3 when compared with that in the naïve group and markedly increased at day 5 when compared with that in the naïve and saline groups (each p < 0.05, **Figure 5C**). On day 3, protein levels of caspase-1 were lower in the RCI001 group than those in the saline group (p < 0.05, **Figure 6C**).

## Discussion

In the present study, we observed that RCI001 inhibited Rac1 activity and various inflammatory cytokines such as IL-1β, IL-6 and TNF-α in LPS-stimulated murine macrophages in a dose-dependent manner. In chemically injured corneas, RCI001 restored corneal epithelial integrity faster than corticosteroid treatment. Activation of the Rac1 and NLRP3 inflammasome/IL-1β axis was suppressed in the RCI001 group when compared with that in the saline group, especially during the early phase of the ocular alkali burn injury. Therapeutic superiority of RCI001 than 1%PDE was also observed when considered the improvement of clinical, histological and molecular signaling aspects.

The Rac1-linked functions of neutrophils and macrophages include phagocytosis, chemotaxis, inflammatory cytokine release, and ROS production through NADPH oxidase activation (18). Additionally, Rac1 is associated with the regulation of the MAPK system, extracellular signal-regulated kinase (ERK), c-Jun N-terminal kinase, Janus kinase (JAK)/signal transducer and activator of transcription (STAT), and the mechanistic target of rapamycin (mTOR) complex (19). Rac1 also controls NF-κB activation and plays a key role in innate immune cell signaling *via* Toll-like receptor (TLR) 2 and 4 (20, 21). On the ocular surface, the surface epithelium critically functions as the first line of defense of the mucosal innate immune system, and an exaggerated epithelial host defense reaction to endogenous bacteria could result in ocular surface inflammation (6). Macrophages on the ocular surface recognize various stimuli *via* TLRs and can induce ocular surface inflammation in several OSDs (6). The NLRP3 inflammasome/IL-1β pathway is activated in OSDs, such as DED and ocular alkali burns, and ROS can activate the NLRP3 inflammasome pathway. Therefore, Rac1 (17) and NLRP3 inflammasome (22) can play important roles to induce pathologic conditions of OSDs. Furthermore, the inflammatory mechanism of OSDs is markedly complex, involving several signal transduction pathways. The simultaneous effects of RCI001 on NLRP3 and Rac1 in OSDs still remain unknown. Therefore, in this study we explored the therapeutic effects of RCI001 on these signaling pathways related complexity in OSDs.

**Figure 7:**
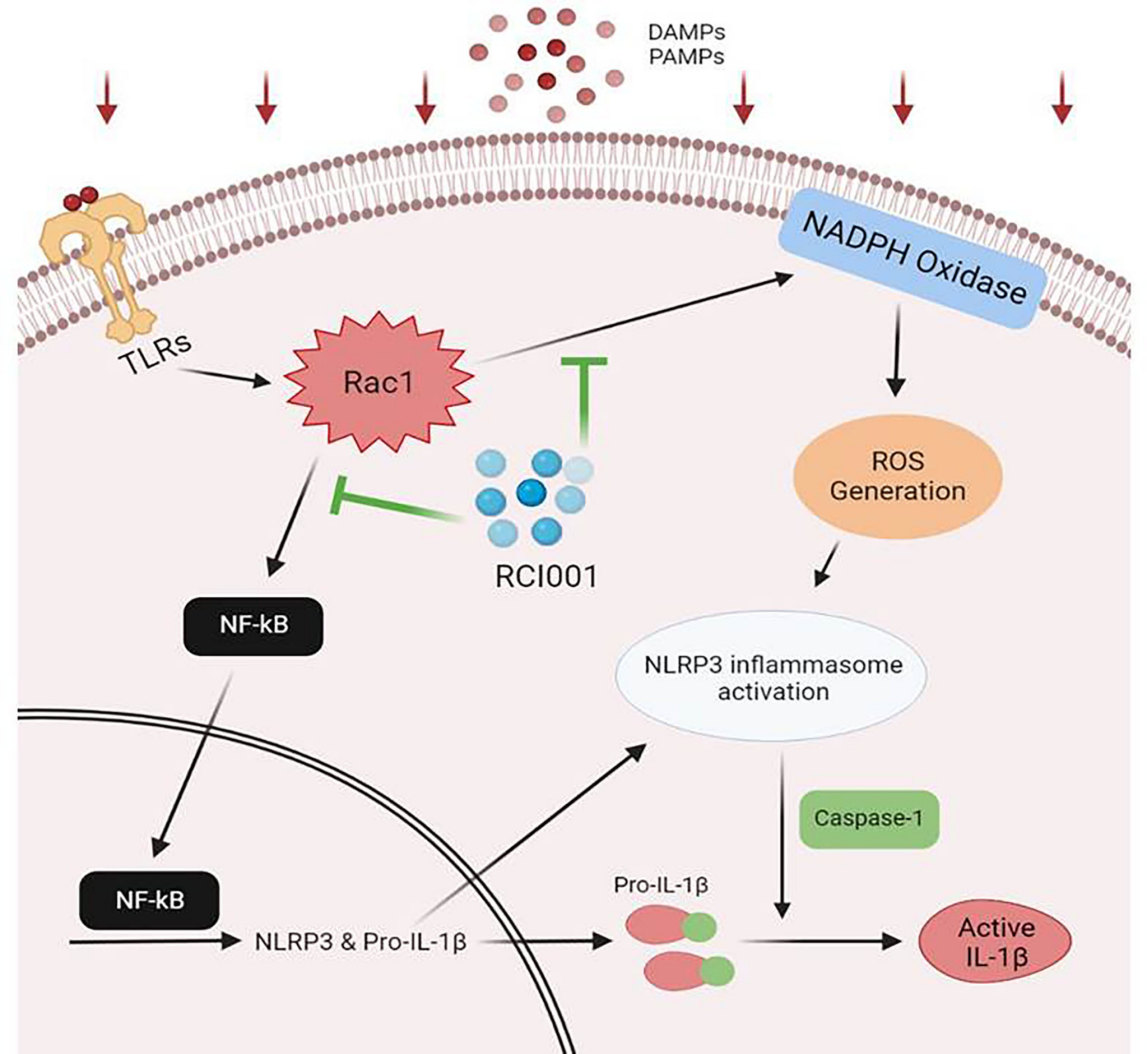
Putative anti-inflammatory mechanism of RCI001 for treating ocular surface diseases.

Previous studies have demonstrated that 8-oxo-dG, the main component of RCI001, inhibits neutrophil and macrophage activation, and 8-oxo-dG is presumed to inhibit Rac1, a G-protein that plays a role in modulating signaling pathways in cellular responses (11, 12). Herein, we revealed that RCI001 inhibited the upregulation of Rac1 and NLRP3 inflammasome/IL-1β pathway, as well as rapidly and effectively restored chemically injured corneal tissues when compared with 1% PDE, the most potent anti-inflammatory agent in commercially available eye drops. Based on our previous and present findings, RCI001 appears to regulate TLR-related inflammatory pathways, NADPH oxidase-related pathways, and Rac1 inhibition. We speculate that the inhibitory effects of RCI001 on Rac1 mediate suppression of the NLRP3 inflammasome/IL-1β pathway, implicated in ROS generation *via* NADPH oxidase or TLR-related NF-κB activation (21, 23) (**Figure 7**, presumed mechanism of RCI001 on the NLRP3 pathway). RCI001 was shown to modulate the activation of MAPK, ERK, and STAT pathways in the ocular alkali burn model (5). In the present study, we observed a discrepancy in the expression of Rac1, NLRP3, and caspase-1 between qPCR and western blotting (**Figures 5**, **6**). Based on our previous studies, it can be speculated that RCI001-induced Rac1 inhibition would occur during the extremely early phase following damage *via* the ROS-NLRP3-IL-1β signaling axis as well as other cytokines like IL-6 and TNF-α. In addition, inhibition of NF-κB activation by RCI001 would simultaneously influence the transcript level of NLRP3 in the nucleus. We postulate that these multimodal actions of RCI001 *via* Rac1 inhibition might explain the observed discrepancy.

To the best of our knowledge, topical corticosteroids are the most potent anti-inflammatory agents known to suppress OSD inflammation. Although T cell inhibitors such as cyclosporin A and tacrolimus can effectively treat OSD, these agents exhibit less potent anti-inflammatory effects than corticosteroids, especially during acute exacerbation of ocular inflammation. Additionally, anti-inflammatory monoclonal antibodies, such as infliximab, adalimumab, tocilizumab, and rituximab, afford limited application against OSDs and remain extremely expensive. We have previously demonstrated the clinical efficacy of corticosteroids in managing DED (24, 25). Nonetheless, the long-term use of topical corticosteroids can induce potential risks such as secondary infection, elevated intraocular pressure, and cataract formation (26). Promoting corneal epithelial healing and modulating ocular surface inflammation are key strategies for treating OSD effectively (6, 7). Notably, topical prednisolone can increase the number of apoptotic cells in the corneal epithelium (27); therefore, the pro-apoptotic effects of corticosteroids should be considered when employed for treating OSD. Increased neutrophil infiltration and ROS activation are reportedly associated with impaired corneal epithelial wound healing (28). RCI001 has potent anti-inflammatory effects similar to 1% PDE and can promote rapid corneal wound healing by suppressing ROS activation and early phase neutrophil and macrophage infiltration into the cornea (5, 16). In this study we found that RCI001 suppressed the over expression of NLRP3, IL-1β, IL6 and TNFα markedly as well as Rac1 thereby accelerating corneal wound healing. Consistently, we have confirmed significant therapeutic effects in environmental- and inflammation-mediated DED mouse models by stimulating tear secretion and modulating inflammation (17). Thus, we speculate that RCI001 could possess superior clinical efficacy than commercially available therapeutics against OSD.

In conclusion, the findings of the present study revealed that topical RCI001 suppressed the expression of activated Rac1 and inflammatory cytokines in LPS-induced macrophages and promptly recovered corneal epithelial integrity by inhibiting the activation of Rac1 and the NLRP3 inflammasome/IL-1β axis in an experimental ocular alkali model. Therefore, topical RCI001 may be a competitive therapeutic for OSDs, promoting corneal epithelial healing and modulating ocular surface inflammation.

## Data Availability Statement

The original contributions presented in the study are included in the article/supplementary material. Further inquiries can be directed to the corresponding authors.

## Ethics Statement

The animal study was reviewed and approved by the Institutional Animal Care and Use Committee of Lee Gil Ya Cancer and Diabetes Institute.

## Author Contributions

Writing – original draft: SK and DK. Writing-review and editing: SK, MR, YK, and DK. Data curation: YJ, Y-AK, and YS. Formal analysis: SK,YJ, and Y-AK. Validation: SK, YJ, and Y-AK. Visualization: SK, YJ, Y-AK, and MR. Methodology: YJ and Y-AK. Project administration: YK and DK. Funding acquisition: YK and DK. Supervision: YK and DK. All authors contributed to the article and approved the submitted version.

## Funding

This research was supported by “Basic Science Research Program” through the National Research Foundation of Korea (NRF) funded by the Ministry of Science and ICT (NRF-2020R1C1C1007372) and “Rediscovery of the Past R&D Result” through the Ministry of Trade, Industry and Energy (MOTIE) and the Korea Institute for Advancement of Technology (KIAT) (Grant No.: 1415170607, P0013936).

## Conflict of Interest

DK owns the patent for the topical use of RCI001 to treat various ocular diseases.

Authors SK, YJ, Y-AK, M-HC, YK, and DK were employed by RudaCure Co., Ltd.

The remaining authors declare that the research was conducted in the absence of any commercial or financial relationships that could be construed as a potential conflict of interest.”

## Publisher’s Note

All claims expressed in this article are solely those of the authors and do not necessarily represent those of their affiliated organizations, or those of the publisher, the editors and the reviewers. Any product that may be evaluated in this article, or claim that may be made by its manufacturer, is not guaranteed or endorsed by the publisher.
